# Validation of a 1DL earliness *per se* (*eps*) flowering QTL in bread wheat (*Triticum aestivum*)

**DOI:** 10.1007/s11032-014-0094-3

**Published:** 2014-05-04

**Authors:** Meluleki Zikhali, Michelle Leverington-Waite, Lesley Fish, James Simmonds, Simon Orford, Luzie U. Wingen, Richard Goram, Nick Gosman, Alison Bentley, Simon Griffiths

**Affiliations:** 1 John Innes Centre, Norwich Research Park, Norwich, Norfolk, UK; 2 The John Bingham Laboratory, NIAB, Huntingdon Road, Cambridge, CB3 0LE UK

**Keywords:** Earliness *per se* (*eps*), Near isogenic lines (NILs), Photoperiod, Vernalization, Wheat

## Abstract

Vernalization, photoperiod and the relatively poorly defined earliness *per se* (*eps*) genes regulate flowering in plants. We report here the validation of a major *eps* quantitative trait locus (QTL) located on wheat 1DL using near isogenic lines (NILs). We used four independent pairs of NILs derived from a cross between Spark and Rialto winter wheat varieties, grown in both the field and controlled environments. NILs carrying the Spark allele, defined by QTL flanking markers *Xgdm111* and *Xbarc62*, consistently flowered 3–5 days earlier when fully vernalized relative to those with the Rialto. The effect was independent of photoperiod under field conditions, short days (10-h light), long days (16-h light) and very long days (20-h light). These results validate our original QTL identified using doubled haploid (DH) populations. This QTL represents variation maintained in elite north-western European winter wheat germplasm. The two DH lines used to develop the NILs, SR9 and SR23 enabled us to define the location of the 1DL QTL downstream of marker *Xgdm111*. SR9 has the Spark 1DL arm while SR23 has a recombinant 1DL arm with the Spark allele from *Xgdm111* to the distal end. Our work suggests that marker assisted selection of *eps* effects is feasible and useful even before the genes are cloned. This means *eps* genes can be defined and positionally cloned in the same way as the photoperiod and vernalization genes have been. This validation study is a first step towards fine mapping and eventually cloning the gene directly in hexaploid wheat.

**Electronic supplementary material:**

The online version of this article (doi:10.1007/s11032-014-0094-3) contains supplementary material, which is available to authorized users.

## Introduction

Genetic variation in emergence and maturation of wheat ears is the consequence of allelic variation at loci controlling the vegetative to floral transition, inflorescence development and stem extension. This variation has major implications for yield potential, abiotic and biotic stress tolerance/avoidance, interactions with agronomic interventions, and our ability for predictive breeding of germplasm adapted to specific environments. The timing of ear emergence is fundamental to plant survival in that it allows plant species to flower at the most suitable period which will allow pollination, seed set and dispersal (Cockram et al. 2007). Three major sets of loci are responsible for the variation in flowering time observed in bread wheat varieties. These loci, which interact with the environment in mediating the transition from vegetative to floral growth in wheat, are as follows: vernalization, photoperiod and the poorly understood earliness *per se* (Herndl et al. 2008; van Beem et al. 2005; Bullrich et al. 2002).

Winter wheat varieties require vernalization (4–8 weeks of cold treatment) before flowering while spring wheat varieties do not. The genetic differences between winter and spring wheat are largely due to allelic variation at the vernalization (*VRN*-*1*) locus (Cockram et al. 2007; Yan et al. 2003). Spring (vernalization insensitive) cultivars have mutations in the promoter or a deletion within the first intron of the *VRN*-*1* genes (Yan et al. 2003).

Photoperiod response in bread wheat is mainly controlled by *Photoperiod*-*1* (*Ppd*-*1*) a pseudo response regulator (*PRR*) gene first identified in barley (Turner et al. 2005) and then the three wheat homoeologous have been identified as *Ppd*-*A1*, *Ppd*-*B1* and *Ppd*-*D1* (Beales et al. 2007; Wilhelm et al. 2009; Díaz et al. 2012). Dominant alleles of these genes make wheat plants photoperiod insensitive, leading to early ear emergence under short days, while those carrying the recessive alleles are very late flowering unless exposed to long days (Worland et al. 1998). Photoperiod and vernalization genes contribute mostly to mega-environment adaptation, and UK wheat varieties are mainly photoperiod sensitive, winter, vernalization requiring types (Griffiths et al. 2009; Worland et al. 1994).

In many UK varieties, the major genes controlling response to vernalization (*VRN*-*1*) and photoperiod (*Ppd*-*1*) are fixed, but breeding populations still segregate widely for ear emergence. The genes responsible for this variation have been categorized as earliness *per se* (*eps*) (Worland et al. 1998) but knowledge of their identities, mechanism and the physiological and agronomic implications of different alleles/allelic combinations are poorly understood.

Earliness *per se* (*eps*) is variation in flowering time revealed when plants have been exposed to adequate vernalization and photoperiod requirements (Appendino et al. 2003). *Eps* loci are defined as the genes that regulate flowering independent of both vernalization or photoperiod environmental cues (Bullrich et al. 2002; Lewis et al. (2008) The *eps* genes are thought to be involved in the fine tuning of wheat flowering time within mega-environments (Griffiths et al. 2009; Valarik et al. 2006) and are responsible for wide adaptation of wheat to different environments (Lewis et al. 2008).

Flowering time QTLs genes are found on almost all the wheat chromosomes (Griffiths et al. 2009) and *ep*s generally causes differences of a few days (1–5) in flowering time (Valarik et al. 2006). *Eps* genes are thought to be involved in different developmental phases including the transition from vegetative to reproductive growth, early and late spike development, stem elongation and heading, which determine grain yield components (Griffiths et al. 2009; Lewis et al. 2008).

Despite its significance, the genetic and physiological basis of *eps* gene function remains largely a matter of conjecture mainly because no *eps* gene has been cloned to date. One *eps* gene that has been studied for about a decade now is the *eps*-*A* ^*m*^ *1* reported to be on the distal region of *Triticum monococcum* chromosome 1A^m^L (Faricelli et al. 2010; Valarik et al. 2006; Bullrich et al. 2002). *Molybdenum Transporter 1* (*MOT1)* and *Filamentation Temperature Sensitive H* (*FtsH4*) have been proposed as candidates for *eps*-*A* ^*m*^ *1*, and tilling work for the two genes is being done to ascertain if one or both, which is a likely candidate (Faricelli et al. 2010). In addition to its effect on heading date, the *eps*-*A* ^*m*^ *1* locus has been reported to be involved in determining the number of spikelets as well as the number of grains per spike in diploid wheat (Lewis et al. 2008).

Hence, understanding the genetics of *eps* and their effect on key traits underlying yield is one avenue that could lead to increased wheat yields. Determining the role of an individual *eps* gene on different wheat developmental phases requires accurate mapping of the gene responsible (Lewis et al. 2008). It is after cloning the gene that it can be studied further, particularly its mechanism of action and how this can be manipulated by wheat breeding. The use of near isogenic lines (NILs) is a step towards fine mapping and eventual cloning of such a gene.

The work described here follows Griffiths et al. (2009), who detected significant heading time QTLs with LOD score greater than 8 on the long arm of chromosome 1DL in four doubled haploid (DH) mapping populations (Spark × Rialto, Charger × Badger, Avalon × Cadenza and Rialto × Savannah) using META QTL analysis. The distal region of chromosome 1DL, a likely orthologue of *eps*-*A* ^*m*^ *1*, had the strongest QTL effect in terms of LOD score, additive effect and stability in the different environments tested (Griffiths et al. 2009). We report here the validation of four independent pairs of NILs segregating for the 1DL QTL (Griffiths et al. 2009) of a cross between Spark (early flowering) and Rialto (late flowering) grown in both the field and controlled environment conditions.

## Materials and methods

### Development of NILs

**Fig. 1:**
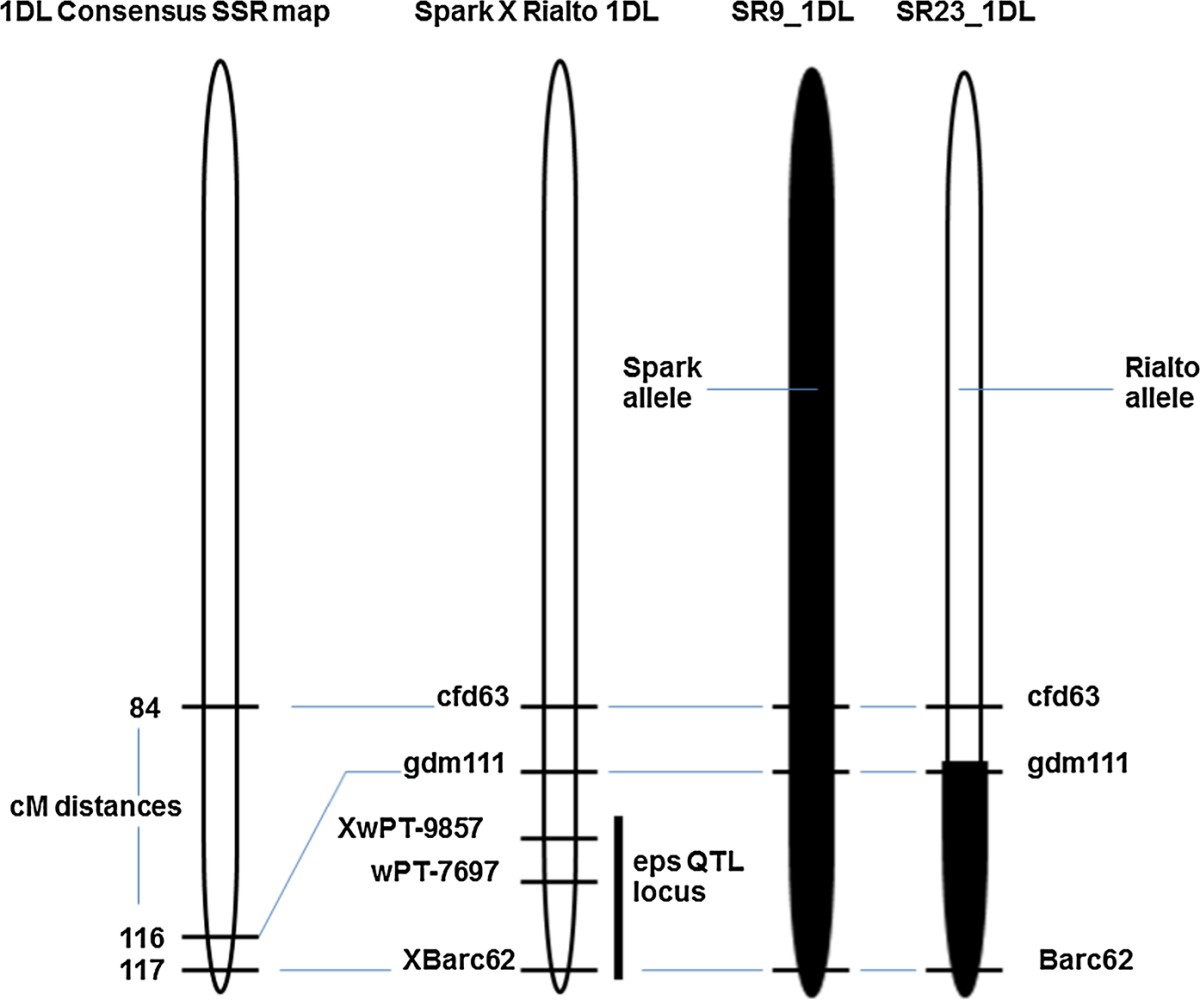
Chromosomal location of markers flanking the ear emergence QTL on 1DL. Xcfd63, Xgdm111 and Xbarc62 were used for the development of Spark × Rialto near isogenic lines derived from SR9 to SR23. The consensus SSR map was adapted from GrainGenes 2.0 database (wheat.pw.usda.gov/). The *eps* QTL locus is adapted from Griffiths et al. (2009)

**Fig. 2:**
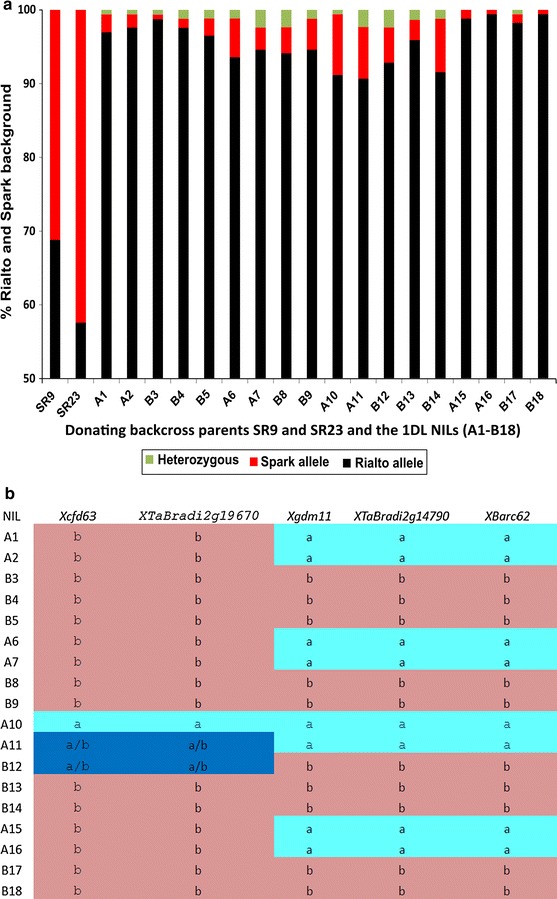
**a** Estimation of the background genotype of the 1DL NILs and the parental donor lines SR9 and SR29 using a total of 173 loci. The average was 95 % Rialto background for all the 18 NILs which is 5 % above the expected 90 % that is theoretically obtained from two backcrosses. NIL pairs A1-B5 and A15-B18 had more than 95 % Rialto background while NIL pairs A6-B14 had Rialto background closer to the expected 90 % Rialto background. **b** The genotype of the 1DL NILs showing that the *eps* effect is distal to the *Xgdm111* marker and that it is the Spark allele which confers early heading. It is also shown here that *TaFT3* a homologue of the barley gene *HvFT3* the suggested candidate for *Ppd*-*2* in barley is not a candidate for the 1DL *eps* effect since all the NILs except one have the Rialto allele at this locus

The background of Spark × Rialto (SR) DH lines was screened using 328 simple sequence repeat (SSR) markers. The lines SR9 and SR23 were selected for use in NIL development because they had relatively higher Rialto background. These two lines also share a common feature of being homozygous for the Spark allele at markers *Xbarc62* and *Xgdm111* (Fig. 1). These markers were reported to be in the QTL interval (Griffiths et al. 2009). The NILs and SR9 and SR23 were also screened using 421 KASPar markers (Allen et al. 2011) and 173 of these were polymorphic and 248 were monomorphic (supplementary Table 1). The 173 polymorphic markers included 34 markers which scored for the Spark allele for SR9 and SR23 selected on all the chromosomes sections that had the Spark allele. Since both of these lines segregated for the early *eps* phenotype, they were ideal as the donating backcross parent with Rialto as recurrent parent because of the higher than 50 % Rialto background (Fig. 2). Both SR9 and SR23 segregated for the early heading phenotype.

Two plants each from SR9 and SR23 (SR9_1, SR9_2, SR23_1, SR23_2) were grown and backcrossed into Rialto to produce backcross 1 (BC_1_) plants. The seeds from BC_1_ plants were grown and then backcrossed into Rialto to produce backcross 2 (BC_2_) heterozygous BC_2_ plants were bagged to enable self-pollination. The BC_2_ plants were screened using markers *Xcfd63, Xgdm111* and *XBarc62* (Fig. 1). For SR23 (SR23_1 and SR23_2), all the plants were fixed for the Rialto allele at marker *Xcfd63* (Fig. 1). Five plants derived from backcrossing SR23_1 consisted of two homozygous for the Spark allele at markers *Xgdm111* and *Xbarc62* and were designated A1 and A2 and three that were homozygous for the Rialto allele designated B3, B4, B5 and these five plants were used as the first NIL validation pair. Four plants derived from SR23_2 formed the second NIL validation pair and were designated A6, A7, B8 and B9 to indicate the NILs homozygous for the Spark and Rialto allele, respectively, at markers *Xgdm111* and *Xbarc62.* Five plants screened from the BC_2_ derived from SR9_1 formed the third NIL validation pair, A10, A11 B12, B13 and B14 and these were homozygous for the Spark and Rialto alleles, respectively, at markers *Xcfd63*, *Xgdm111* and *Xbarc62*. The fourth NIL validation pair was screened from BC_2_ plants derived from SR9_2 and this comprised of A15, A16, B17 and B18, and these were all fixed for the Rialto allele at *Xcfd63* but homozygous for the Spark and the Rialto alleles, respectively, at markers *Xbarc62* and *Xgdm111*.

### Validation of *eps* using four Spark × Rialto NILs

The experiment was divided into field and controlled environments. For the controlled environment, the plants were sown in December 2011, fully vernalized under short days (10-h light) for eight weeks at 6–10 °C using natural vernalization in an unheated glasshouse. The plants were then grown at 13–18 °C under short days (SD, 10-h light), long days (LD, 16-h light) and very long days (VLD, 20-h light) using movable benches set to give the SD, LD and VLD photoperiods. Additional lighting was provided using 4-h and 8-h artificial white light (tungsten bulbs) to aid the LD and VLD, respectively. We used eight 60 W tungsten lamps in each of LD and VLD treatments spaced 90 cm apart and 2.1 m 1 above the bench on which the plants were growing. This delivers 1 micromole s^−1^ m^−2.^

For the controlled environment treatment, five plants were grown in 1 litre pots for each NIL in each photoperiod treatment. We used the randomized complete block design from EDGAR II experimental design generator and randomizer software (http://www.edgarweb.org.uk/) designed by James KM Brown John Innes Centre. Differences in flowering time between the NIL pairs was determined at growth stage 55 (GS55) according to the scale by Zadoks et al. (1974). The Student’s *t* test was used to test for significance between the heading date means of the NIL pairs. Five plants each of the wheat cultivars Spark, Rialto, Claire, Malacca and Hereward were grown as controls to determine whether the plants had been adequately vernalized. Díaz et al. (2012) reported that Hereward flowered more than 30 days later than Malacca and Claire when inadequately vernalized (4 weeks) and this was associated with copy number variation at *Vrn*-*A1*. All plants in this study were vernalized for 8 weeks at 6–10 °C and then grown under SD, LD and VLD. Ear emergence for Spark, Rialto, Claire, Malacca and Hereward was scored the same way as for the NILs.

Field plots were sown in September 2010 at Church Farm Norwich, Norfolk. The NIL pairs were drilled in randomized 1 m^2^ plots, and three randomized plots were grown for each NIL. Plants were then scored for ear emergence the same way as the controlled environment plants were scored. Plants were naturally vernalized in the field over winter. The differences in heading date between the NILs were scored as days to heading after 1 May 2011 for the whole population.

### Sequencing *TaFT3*-*D1* the homologue of the barley gene *HvFT3*

#### Homology searching the “Chinese Spring” unassembled reads database

The barley gene *HvFT3* has its homologue on wheat 1DL. We wanted to determine whether this gene could be the candidate for the wheat 1DL *eps* effect. Since our study was being carried out using hexaploid wheat, we assembled the three *TaFT3* gene homoeologous (A, B and D) from the unassembled reads of the Chinese Spring sequence database (Brenchley et al. 2012) to enable us to design primers which were specific to 1DL. The mRNA sequence of barley gene *HvFT3* accession number DQ411319.1 (Faure et al. 2007) was used to homology search the “Chinese Spring” unassembled 454 reads database using the **B**asic **L**ocal **A**lignment **S**earch **T**ool (BLASTn) algorithm (Altschul et al. 1990). The three wheat homoeologous of the gene were then assembled using vector NTI sequence alignment tool. Homoeologous single nucleotide polymorphisms between the putative three homoeologous allowed us to identify the three homoeologou which we designated *X*, *Y* and *Z* at this point.

### Identification of the A, B and D homoeologous of *TaFT3* using the *Aegilops tauschii* and *Triticum urartu* unassembled reads database

One of the putative A, B and D genome homoeologous designated *X*, *Y* and *Z* was used to blast search the *A. tauschii* sequence database (You et al. 2011) and the *A. tauschii* sequences were assembled and aligned with the three putative wheat homoeologous *X*, *Y* and *X*. The D genome from the putative wheat homoeologous had the highest sequence identity match with the *A. tauschii* sequence. Furthermore, the SNPs that were specific to the D genome matched the *A. tauchii* sequences and hence enabled accurate assigning of the 1DL sequences. Although it was not essential to our work, we also were able to distinguish the A and the B sequences by using the *Triticum urartu* sequence database (Ling et al. 2013) to identity the A genome and the remaining sequence was then assigned to the B genome.

### Standard PCR protocol

The PCR were done as described by Díaz et al. (2012) with a few modifications. The PCR was carried out in 20 µl reactions comprising of 2.5 µl of 20 ng/µl genomic DNA dissolved in 1× TE buffer, 0.4 µl of 10 mM dNTPs (Promega UK LTD) dissolved in 1× TE buffer, 1.6 µl of 25 mM MgCl_2_, 4.0 µl of 5× clear buffer, 1 µl each of 5 µM (dissolved in 1× TE buffer) forward and reverse primers, 0.080 µl GO TAQ FLEXI DNA (Promega UK LTD) polymerase (5U/µl), 9.42 µl of double distilled water.

### PCR conditions

The PCR had 40 cycles and 55 °C was the annealing temperature. The first step was initial denature done at 95 °C for 2 min. Forty cycles involved denaturation for 20 s at 95 °C, annealing at 55 °C for 20 s and polymerization at 72 °C for 1 min per kb. After the forty cycles, the PCR was held at 72 °C for 5 mins and then kept at 10 °C until removal to a freezer or analysis on agarose gel.

### Sequencing of genes on 1DL

The assembled genes were used to design a series of overlapping PCR amplicons spanning the entire *TaFT3-D1* gene using the method described by Díaz et al. (2012). Amplicons were obtained from genomic DNA using the standard PCR protocol and were directly sequenced using ABI Big Dye Mix v3.1 (Applied Biosystems Inc) under the manufacturer’s conditions, with products resolved on an ABI 3730 capillary electrophoresis instrument. The primers amplified PCR fragments ranging in size from 400 to 1500 bases from both Spark and Rialto.

### Scoring single nucleotide polymorphisms (SNPs) in *TaFT3*-*D1*

 Scoring of SNPs in *TaFT3*-*D1* was done as described for *Vrn*-*A1* by Díaz et al. (2012). We used KBioscience KASP reagents (www.kbioscience.co.uk) in reactions containing distilled water (2 µl), KASPar mix (4 µl), primers (0.1 µl), 50 mM MgCl_2_ 0.064 ml) and DNA (2 µl). An activation time (94 °C, 15 min) was followed by 20 cycles of 94 °C for 10 s; 57 °C for 5 s; 72 °C for 10 s followed by 24 cycles of 94 °C for 10 s; 57 °C for 20 s; 72 °C for 40 s. Fluorescence was read as an end point reading at 25 °C. Primer combinations were; Exon4_A/G SNP specific primers: gaaggtgaccaagttcatgctAGGCGGAAGAAGGTTTAG**A** gaaggtcggagtcaacggattGGCGGAAGAAGGTTTAG**G** (0.16 mM). Generic primer ATGGTCAGTACTCTGTACTATCTAGTCC (0.4 mM).

## Results

**Fig. 3:**
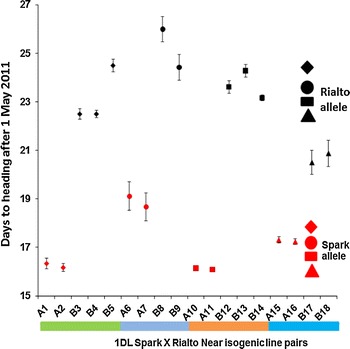
GS55 for leading tillers of field grown (UK) Spark × Rialto NILs. The NILs with the Spark allele are consistently early (*red*). These are four independent NIL pairs showing consistent segregation of early and late phenotypes (*p* value <0.0001). The *red* and *black colours* are used for the NILs carrying the Spark and Rialto alleles at 1DL, respectively. The different shapes are used to distinguish between NIL pairs. *Diamonds* are used for the first NIL pair (A1-B5), *circles* are used for the second NIL pair (A6-B9), *rectangles* are used for the third NIL pair (A10-B14), and *triangles* are used for the fourth NIL pair (A15-B18). The *vertical bars* on the shapes are the standard error of the mean

**Fig. 4:**
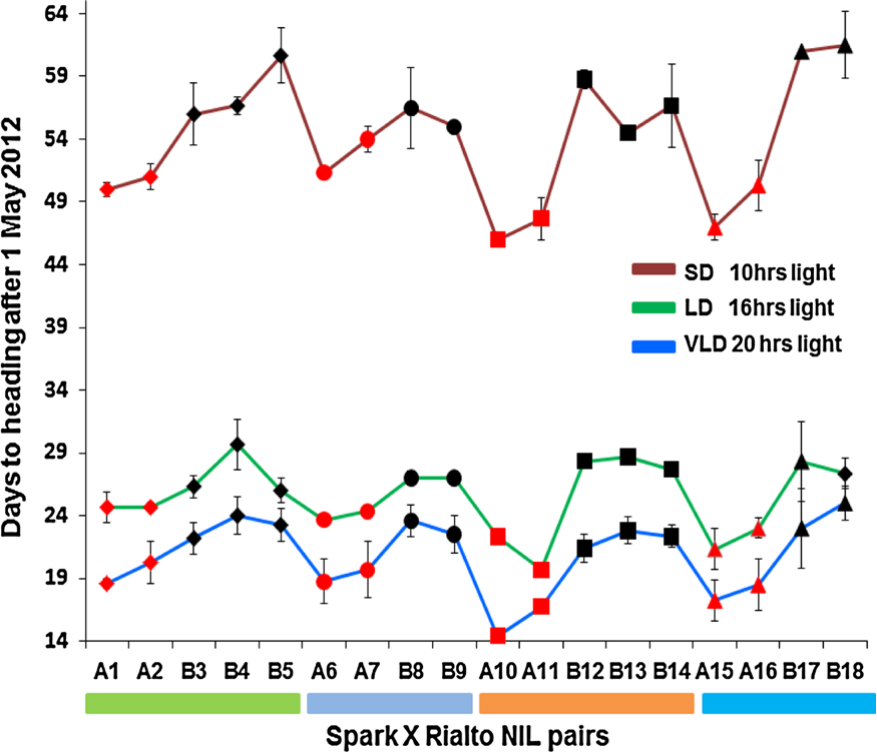
GS55 for leading tillers of controlled environment grown Spark × Rialto NILs. The *red* and *black colours* are used for the NILs carrying the Spark and Rialto alleles at 1DL, respectively. The different shapes are used to distinguish between NIL pairs. *Diamonds* are used for the first NIL pair (A1-B5), circles are used for the second NIL pair (A6-B9), *rectangles* are used for the third NIL pair (A10-B14), and *triangles* are used for the fourth NIL pair (A15-B18). The *vertical bars* on the shapes are the standard error of the mean. The *red green* and *blue lines* connecting the symbols are for the purpose of distinguishing the three photoperiod treatments

In the previous study (Griffiths et al. 2009), the 1DL QTL was detected by the analysis of DH lines grown in the filed only. In contrast, the current study developed and evaluated NILs grown under controlled environments and in the field (Figs. 1, 2, 3, 4). In both field and controlled environments, the NILs carrying the spark 1DL segment are consistently early flowering than the Rialto (Figs. 2b, 3, 4). We checked the statistical significance of our results using the Student’s *t* test for both field and controlled environment grown NILs (Table 1). All NIL pairs had significant differences in mean heading date in both the field and controlled environments except NIL pair two (A6, A7, B8 and B9) which had a non-significant *p* value under short days but significant *p* values for LD and VLD (Table 1).

**Table 1:** Mean heading date after 1 May and Student’s *t* test values of 1DL NILs grown in the field and controlled environments

NIL pairs	1DL QTL interval genotype	Field		Controlled environment					
				VLD (20-h light)		LD (16-h light)		SD (10-h light)	
		Mean Heading date	Student’s *t* test *p* value	Mean	Student’s *t* test *p* value	Mean	Student’s *t* test *p* value	Mean	Student’s *t* test *p* value
A1, A2	Spark	16.3	0.0001	19.3	0.0037	24.3	0.032	50.5	0.012
B3, B4, B5	Rialto	23.3		23		26.1		56.4	
A6, A7	Spark	18.9	0.0001	19.1	0.035	24	0.0001	52.4	0.2
B8, B9	Rialto	25		23.3		27		54	
A10, A11	Spark	16.1	0.0001	15.4	0.001	21	0.0001	46.8	0.0001
B12-14	Rialto	23.7		22.2		28.2		56.8	
A15, A16	Spark	17.3	0.0001	17.9	0.0028	22	0.0001	48.7	0.0002
B17, B18	Rialto	20.7		24.3		27.8		60.2	

The mean heading date are the average days to ear emergence of NILs carrying the Spark or Rialto allele at 1DL for each NIL pair. The ear emergence was measured at GS55 using the scale by Zadoks et al. (1974)

Our results therefore validate the existence and chromosome position of the flowering time QTL as marker assisted introgression of the Spark 1DL region caused early flowering in the relatively late flowering Rialto background (Figs. 2, 3, 4). The results also show that the 1DL heading QTL is an *eps* effect given that the NILs with the Spark allele are early flowering independent of photoperiod (Fig. 4). This was suggested but not proven by the field study of Griffiths et al. (2009).

### Genotype of 1DL NILs at loci known to regulate heading date in Spark × Rialto

The NILs used in this study were created with the recurrent parent Rialto. In cases were the donor parent carried Rialto alleles, there is no issue with potential Spark background effects in the BC2 NILs. The presence of Spark alleles is of particular importance in the regions where we know heading date QTLs are likely to segregate. So, we checked the genotype of the NILs at ear emergence QTL loci on 1BL, 2A, 3A, 3B, 4B, 4D, 5AL 5B, 6A, 6B, 7A and 7D since Spark and Rialto were reported to segregate for ear emergence at these loci (Griffiths et al. 2009). The donor parent SR9 was already fixed for Rialto chromosome arms 3B, 4B and 4D, 5B hence the NILs A10, A11, B12, B13, B14, A15, A16, B17, B18 developed from SR9 were all fixed Rialto at 3B, 4B and 4D since Rialto was the recurrent parent. We confirmed this when we genotyped the NILs (Supplementary Table 1). SR23, the other donating parent, had fixed Rialto chromosome arms at 1B, 3A, 4B, 5B hence the NILs, A1, A2, B3, B4, B5 A15, A6, A7, B8 and B9 were all fixed Rialto at 1B, 3A and 4B since Rialto was the recurrent parent for backcrossing. We also confirmed this when we genotyped the NILs using KASPAr markers (Supplementary Table 1).

The recurrent parent SR9 had Spark chromosome arms at 1B, 2A and 3A, 6AL while the recurrent parent SR23 had Spark chromosome arm at 2A, 4D, 6AL while chromosome. We also checked these loci as they were likely to cause some background noise if these areas were fixed with spark alleles in the background of the NILs. At 1B, NILs A10, A11, B12, B13 and B14 had the Spark allele while the rest had the Rialto allele (Supplementary Table 1). At 2A, NILs A6 and B9 had Spark alleles in the QTL region while the rest had Rialto alleles. At 3A, NILs A10, A11, B12, B13 and B14 had the Spark allele while the rest had Rialto. At 4D, all the NILs had the Rialto allele. At 5AL, NILs A6, A7, B8 and B9 were heterozygous but the rest were fixed Rialto. All the NILs were fixed Rialto at 5BL, 6B and 7D while NILs A7 and B8 had Spark alleles and NILs B9 was heterozygous at 7A and the rest were fixed Rialto. (Supplementary Table 1). These differences in the NILs background can be speculated to account for some of the variations in heading observed between the NILs.

### Differences in heading date among NILs

**Fig. 5:**
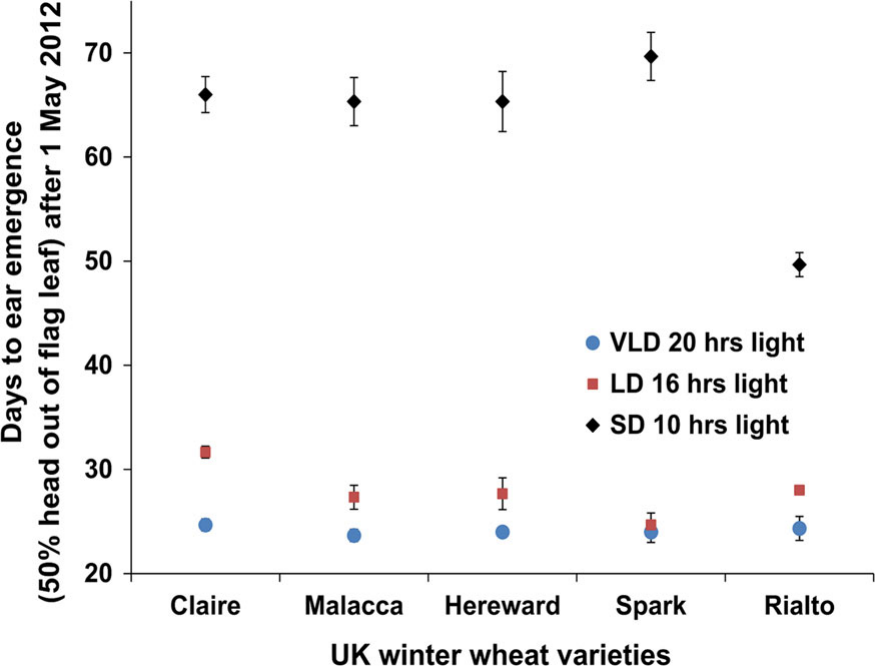
GS55 of controlled environment grown elite UK wheat varieties. Rialto is relatively earlier flowering under short days than the rest of the varieties which flower almost at the same time under short days except Spark which is slightly late. The *vertical bars* on the shapes are the standard error of the mean

Our results show that there are some differences in heading date among the Nils (Figs. 3, 4) but these differences are less in the parental lines (Fig. 5). This maybe speculated to be due to different backgrounds between the Nils and parental lines. The parental lines are more homogenous than the NILs which have heterozygous segments (Supplementary Table 1). Furthermore, we also show that NIL pair 2 (A6, A7, B8 and B9) is heterozygous at 5AL loci and this NIL pair is the only one which had non-significant *p* value under short days (Table 1). Is possible that there could be a short day effect at this locus which interacts with the 1DL locus given that the 5AL locus is linked to the *XBarc 151* marker (data not shown) which is known to be linked to genes that affect flowering time such as *Vrn*-*A1, PHYC* (Díaz et al. 2012; Distelfeld and Dubcovsky 2010).

We also carried out an analysis of variance (ANOVA) to determine whether there was an interaction between the 1DL genotype and photoperiod, but there was no significant interaction between 1DL genotype and photoperiod (*p* = 0.10851) and the F value was 2.2516 which was less than the *F* critical value 3.051.

### Possibility of *TaFT3* as a candidate for 1DL

Our results (Fig. 2b) show that *Triticum* *aestivum* *FLOWERING LOCUS T 3* (*TaFT3*), the wheat homologue of barley gene *FLOWERING LOCUS T 3* (*HvFT3*), a candidate for *PHOTOPERIOD H2* (*Ppd*-*H2*) (Faure et al. 2007), is not a candidate for the 1DL *eps* effect. A single nucleotide polymorphism (SNP) in exon 4 which is a silent mutation in the D copy of *TaFT3* (*TaBradi2g19670*) allowed us to develop a KASPAr marker (X*TaBradi2g19670*) which distinguishes Spark (accession number KJ661739) from Rialto (accession number KJ661740). All the NILs at this locus have the Rialto allele except NIL10, 11 and 12 (Fig. 2b). NILs 11 and 12 have both alleles of *TaFT3* (Fig. 2b) but NIL 11 is early flowering relative to NIL 12 (Figs. 3, 4). Given that all the early flowering NILs have the Spark allele at *Xgdm111* (Fig. 2b), we conclude that the 1DL *eps* effect is downstream of *TaFT3* and hence *TaFT3* is not a candidate for the 1DL *eps* effect.

It is also shown that the NILs segregate for ear emergence when fully vernalized for eight weeks (Fig. 4). A recent report by Díaz et al. (2012) showed that wheat segregates for heading when inadequately vernalized (less than 8 weeks) and grown under LD. Our study used Claire, Malacca and Hereward which require short, intermediate and long exposure to vernalization, respectively, as controls (Fig. 5). Hereward flowers more than 30 days later than Claire and Malacca when inadequately vernalized for four weeks (Díaz et al. 2012). In the current study, the three varieties all flower at the same time when vernalized for 8 weeks, particularly under SD and VLD, with Claire (which is earliest flowering when inadequately vernalized) flowering 5 days later than the other two under long days (Fig. 5) showing that we had exposed our experiment to adequate vernalization, hence the segregation of the NILs can be attributed to an *eps* gene(s). There is also a separate effect that causes Rialto to be early flowering under short days and the 1DL effect is independent of this (Fig. 5).

## Discussion

Our results also show that the NILs have more than 90 % Rialto background (Fig. 2), and the average for the 18 NILs was 95 %. The expected Rialto background from two backcrosses is about 90 % given that we started with around 60 % Rialto background for the donating parents SR9 and SR23 (Fig. 2). This result is most likely due to the random nature of recombination. However, one possible explanation is that most of the D chromosomes had very few markers which may have underestimated the Spark background. Another possible explanation is that even though the 173 markers were selected to represent as much of the chromosomes as possible, they are not adequate to give an accurate estimation given the big size of the bread wheat genome. The markers that had scored for the Spark allele for SR9 and SR23 were used to score the NILs and most of them showed that the NILs had the Rialto alleles at these loci suggesting that the background was indeed near isogenic. Spark and Rialto also have close lineage from their pedigrees and the 248 monomorphic markers from a total of 421 (data not shown) also shows their close relatedness. Taken all together, our results suggest that the background of the NILs was very similar.

It is interesting to note that the NIL pairs have the same alleles in the background whether it is Spark or Rialto hence comparing the NIL pairs at1DL should give comparable results. However, there are some minor differences within the NILs themselves, for example, NIL B5 flowering 2 days later than NILs B3 and B4 and NIL B8 and B9 have a difference in 2 days in the field. It is possible that there are other QTL that could not be detected by the DH lines whose effect is now observable. Again the KASPAr markers we used cannot detect copy number variations which could be causing these differences in heading within NILs.

The *eps* effects are important adaptive traits but they have not been well studied in the past. It was reported almost half a century ago that earliness *per se* genes caused some photoperiod sensitive varieties to flower earlier than photoperiod insensitive varieties (Martinić 1975) but the genes responsible are still not characterized. The introduction of *eps* effects into UK germplasm in the 1980s resulted in an accelerated flowering of those varieties, which also significantly increased yield relative to earlier varieties (Austin et al. 1980). One reason why *eps* genes have not been well studied is that they were often mapped in crosses segregating for *Ppd and Vrn*, which usually mask the *eps* effects. Worland et al. (1994) underscored the need to develop genetic stocks that could be used to reveal the importance of *eps* in wheat adaptability. The NILs developed and described here are valuable genetic stocks to study *eps* and lay a foundation for unravelling their effects and may also be useful in breeding programmes.

Our work follows Griffiths et al. (2009) who carried out META QTL analysis using doubled haploid populations and suggested that there was an *eps* effect on the distal end of chromosome 1DL. An aim of this study is to contribute towards fine mapping of the gene, and the validation of NILs segregating for the QTL is a necessary first step. A 20-h photoperiod satisfies the photoperiod requirements of most photoperiod sensitive wheat given that wheat is a long day plant (flowering rapidly in long days of about 16-h light but very late in short days of about 10-h light), unless they carry photoperiod insensitive *Ppd*-*1a* alleles (Beales et al. 2007; Wilhelm et al. 2009; Díaz et al. 2012).

The wheat varieties used in this study are UK winter wheat varieties which are photoperiod sensitive (Worland et al. 1998). The eight weeks vernalization treatment satisfied the vernalization requirement given that Hereward, which flowers very late relative to Malacca when inadequately vernalized for four weeks (Díaz et al. 2012), flowered at the same time as Malacca when vernalized for eight weeks (Fig. 5). Since the segregation of the NILs cannot be accounted for by photoperiod or vernalization requirements, it falls in the *eps* group of genes that affect flowering (Bullrich et al. 2002; Appendino et al. 2003; Lewis et al. 2008) possibly through other developmental pathways.

The segregation of the NIL pairs, which have a common background, should enable further study to determine the genetic basis of *eps* gene(s) as the region that is defined by the flanking markers used to develop the NILs is known. The study included both field grown and controlled environment grown material, and both environments gave consistent results where the NILs carrying the Spark allele were early flowering relative to those with the Rialto.

A similar *eps* study done using *T. monococcum* (Lewis et al. 2008), suggested that the gene responsible had pleiotropic effects on spikelet number and grains per spike in addition to the heading time effect. Griffiths et al. (2009) suggested but did not prove that the same gene was likely responsible for *eps* in both *T. monococcum* and *T. aestivum*. Successful cloning of the gene represents a potential step towards increasing yield, because the delicate combination of genes responsible for grain size and spikelet number would eventually lead to overall yield increase. This validation study is a step towards cloning the gene and fine tuning flowering adaptation in wheat.

An important question which remains unanswered from our work is whether the 5 days difference in flowering time will cause a significant yield difference between the two NIL pairs and if that will be dependent on variable environments. If the 1DL *eps* effect is yield neutral, the two alleles can be used to breed wheat for an environment that requires earlier flowering (Spark allele) to avoid stress such as late drought, or an environment that is favourable to late flowering wheat (Rialto allele) to take advantage of a long growing season, without a significant yield penalty. The validation of grain yield of the NILs will answer this question.

The genes *MOT1* and *FtsH4*, the proposed candidates for *eps*-*Am1*, (Faricelli et al. 2010) are both likely candidates for 1DL QTL since both genes are downstream of *Xgdm111*. Another gene *T. aestivum* *EARLY FLOWERING 3* (*TaELF3*) a homologue of the barley gene *EARLY MATURITY 8* (*eam8*)*/mat*-*a* (Faure et al. 2012; Zakhrabekova et al. 2012) is also a possible candidate given that is also downstream *of Xgdm111*. The near isogenic lines we report here cannot enable the separation of the three genes to identify a possible candidate. Ongoing and current work is using a recombinant population between Spark and Rialto to fine map and eventually clone the candidate gene. However, we affirm that *TaFT3* is not a candidate for the 1DL QTL.

## Electronic supplementary material

Below is the link to the electronic supplementary material.

The genotype of the NILs at loci known to segregate for flowering time in Spark × Rialto DH population. All the KASPAr markers are from Allen et al. 2011. The KASPAr markers were performed in duplicate. (XLSX 54 kb)
